# Short-chain fatty acids can improve lipid and glucose metabolism independently of the pig gut microbiota

**DOI:** 10.1186/s40104-021-00581-3

**Published:** 2021-05-06

**Authors:** Hua Zhou, Bing Yu, Jing Sun, Zuohua Liu, Hong Chen, Liangpeng Ge, Daiwen Chen

**Affiliations:** 1 Key Laboratory of Animal Disease-Resistance Nutrition, Chengdu, 611130 Sichuan China; 2 Animal Nutrition Institute, Sichuan Agricultural University, Chengdu, 611130 Sichuan China; 3 Key Laboratory of Pig Industry Sciences, Rongchang, 402460 Chongqing China; 4 Chongqing Academy of Animal Sciences, Rongchang, 402460 Chongqing China; 5 College of Food Science, Sichuan Agricultural University, Ya’an, 625014 Sichuan China

**Keywords:** Germ-free, Glucose metabolism, Lipid metabolism, Pig model, Short-chain fatty acids

## Abstract

**Background:**

Previous studies have shown that exogenous short-chain fatty acids (SCFAs) introduction attenuated the body fat deposition in conventional mice and pigs. However, limited studies have evaluated the effects of exogenously introduced SCFAs on the lipid and glucose metabolism independently of the gut microbiota. This study was to investigate the effects of exogenous introduction of SCFAs on the lipid and glucose metabolism in a germ-free (GF) pig model.

**Methods:**

Twelve hysterectomy-derived newborn pigs were reared in six sterile isolators. All pigs were hand-fed with sterile milk powder for 21 d, then the sterile feed was introduced to pigs for another 21 d. In the second 21-d period, six pigs were orally administrated with 25 mL/kg sterile saline per day and considered as the GF group, while the other six pigs were orally administrated with 25 mL/kg SCFAs mixture (acetic, propionic, and butyric acids, 45, 15, and 11 mmol/L, respectively) per day and regarded as FA group.

**Results:**

Orally administrated with SCFAs tended to increase the adiponectin concentration in serum, enhance the CPT-1 activity in longissimus dorsi, and upregulate the *ANGPTL4* mRNA expression level in colon (*P* < 0.10). Meanwhile, the mRNA abundances of *ACC*, *FAS*, and *SREBP-1C* in liver and *CD36* in longissimus dorsi of the FA group were decreased (*P* < 0.05) compared with those in the GF group. Besides, the mRNA expression of *PGC-1α* in liver and *LPL* in longissimus dorsi tended to (*P* < 0.10) upregulate and downregulate respectively in the FA group. Moreover, oral administration of SCFAs tended to increase the protein level of GPR43 (*P* < 0.10) and decrease the protein level of ACC (*P* < 0.10) in liver. Also, oral administration of SCFAs upregulated the p-AMPK/AMPK ratio and the mRNA expressions of *GLUT-2* and *GYS2* in liver (*P* < 0.05). In addition, the metabolic pathway associated with the biosynthesis of unsaturated fatty acids was most significantly promoted (*P* < 0.05) by oral administration of SCFAs.

**Conclusions:**

Exogenous introduction of SCFAs might attenuate the fat deposition and to some extent improve the glucose control in the pig model, which occurred independently of the gut microbiota.

**Supplementary Information:**

The online version contains supplementary material available at 10.1186/s40104-021-00581-3.

## Background

Emerging evidence indicated that gut microbiota plays a critical contributor to the host’s health [1]. Noteworthy, the beneficial effects of gut microbiota are at least partly attributed to the short-chain fatty acids (SCFAs), which are the end-products produced from the fermentation of dietary fiber and resistant starch [2, 3]. SCFAs acts as pivotal roles in various biological activities, such as lipid and glucose metabolism [4–6]. Recent studies have reported that oral administration of SCFAs increased the energy expenditure and fat oxidation in obese mice [7], prevented body weight gains, and enhanced insulin sensitivity in high-fat diet-fed mice [8]. Moreover, exogenously introduced SCFAs reduced the fat deposition in weaned and growing pigs by decreasing lipogenesis and promoting lipolysis in different tissues [9, 10]. Human intervention reports found that SCFAs could regulate whole-body substrates and energy metabolism by increasing fasting fat oxidation and resting energy expenditure [11]. However, controversy still exists considering the role of SCFAs in lipid metabolism. Previous work demonstrated that SCFAs were thought to contribute additional calories in the obese, thus resulting in additional weight gain [12]. Conflicting literature indicated that enhanced acetate turnover aggravated the development of obesity and insulin resistance in rodents [13]. The inconsistent effects of SCFAs on lipid and glucose metabolism might affect by gut microbiota, which plays a vital role in the development and progression of obesity [14, 15]. It has been observed that the *Christensenella* genus could prevent weight gain in mice [16], and the *Akkermansia* genus was reported to correlate with lower visceral fat deposits [17]. Decreased the abundances of *Bacteroides* and *Prevotella* were indicated positively correlated with energy intake and adiposity [18]. Moreover, the numbers of microbiota are closely associated with SCFAs concentrations [19]. Thus, gut microbiota may interfere with the effects of exogenous introduction of SCFAs on the host’s health, and further well-controlled studies are urgently needed. Germ-free (GF) animals are free from living microorganisms, including bacteria, viruses, fungi, protozoa, and parasites throughout their life, and are reared in sterile environments [20, 21]. The domestic pig (*Sus scrofa*) is a preferable model of human health, which is similar in anatomy, physiology, and genetics to humans [22, 23]. Accordingly, the pig with an absence of gut microbes is the valid experimental model for dissecting the effects of exogenously introduced SCFAs on lipid and glucose metabolism. Moreover, the systematic crosstalk of exogenous SCFAs and the host’s lipid and glucose metabolism has been rarely investigated in the absence of gut microbiota. Therefore, this study was to take biochemistry and metabolomics analysis to explore the effects of oral administration of SCFAs on the lipid and glucose metabolism in a GF pig model, which may help us to further understand the underlying mechanisms of SCFAs for alleviating fat deposition and promoting the host health.

## Materials and methods

The experiment was conducted at the Experimental Swine Engineering Center of the Chongqing Academy of Animal Sciences (CMA No. 162221340234; Chongqing, China).

### Animals

Twelve neonatal GF pigs were delivered via hysterectomy from four multiparous Bama sows (a native breed of China). On 112 d of gestation (full-term, 114 d), pregnant sows were anesthetized with 4% isoflurane, and the uterus was excised from the sow and transferred into a sterile isolator (DOSSY Experimental Animals Co., Ltd., Chengdu, China) through a tank containing 120 L of 0.1% peracetic acid for decontamination. Then, 12 neonatal pigs (male and female in half) were taken from the uterus and transferred to six rearing isolators (Class Biologically Clean Ltd., Madison, WI, USA). The rearing isolator contains a checkboard, two pigs per isolator, and fed separately. The rearing isolators had been performed by spraying with 1% peracetic acid in advance and preserved in sterile environments as described previously [21]. The pig’s skin, sterile environments, rectal swabs, and oral mucosa were detected via anaerobic (thioglycollate medium) and aerobic (brain heart infusion broth) culture of samples at least every week in accordance with the Chinese National Standard (GB/T 14926-41-2001). Colonic digesta was collected at the end of the experiment for further confirmation of sterile status.

### Experimental design and diet

Among the six rearing isolators, three of them were designated as the FA group, and the other three isolators were treated as the GF group. These pigs in the FA and GF groups were hand-fed ^60^Co-γ-irradiated sterile milk powder (Table S1) and diluted with sterile water 1:4 for 21 d. A corn-soybean feed (Table S2) was formulated according to the requirements of Chinese Feeding Standards (2004) for local pigs. It was sterilized by Co60-γ-radiation and introduced to the GF and FA pigs for another 21 d. In the second 21-d period, the FA group was orally administrated with sterile 25 mL/kg SCFAs mixture (acetic, propionic, and butyric acids, 45, 15, and 11 mmol/L, respectively) per day, and the GF group was orally administrated with 25 mL/kg sterile saline per day. The introduction volume of SCFAs mixture or sterile saline for each pig per day is presented in Table S3. Furthermore, the SCFAs mixture was confected in the laminar airflow clean benches, and the acetic, propionic, and butyric acids (analytically pure) was filtered through a 0.22-μm membrane and mixing with sterile water. During the two 21-d periods, pigs were allowed ad libitum access to sterile water. To maintain the sterile environment in the present study, when the SCFAs, water, milk, and feed in the rearing isolators were consumed, the replacement containers for sterile SCFAs, water, milk, and feed were delivered into the rearing isolator via the transfer port. Before transferred into the transfer port, the containers were preliminarily decontaminated with 0.5% peracetic acid and then spraying with 1% peracetic acid.

### Sample collections

In the morning of d 42, blood was drawn from anterior vena cava, centrifuged at 3,000×*g* for 15 min, and stored at − 80 °C for further measurements. After blooding, pigs were euthanized by isoflurane anesthesia. The abdomen was opened in the laminar airflow clean benches, and the samples of the colon, liver, and longissimus dorsi were immediately collected in liquid nitrogen and stored at − 80 °C for further analysis.

### Serum parameters measurement

The concentrations of adiponectin, insulin, glucagon, glucagon-like peptide 1, and leptin in serum were detected by commercial enzyme-linked immunosorbent assay (ELISA) kits from Chenglin Co. Ltd. (Beijing, China) in accordance with the manufacturer’s instructions. The levels of total cholesterol (TC), triglyceride (TG), high-density lipoprotein-cholesterol (HDL-c), low-density lipoprotein-cholesterol (LDL-c), and glucose in serum were measured using commercial kits (Nanjing Jiancheng Bioengineering Institute, Nanjing, China) following with the manufacturer’s instructions. Each parameter was simultaneously measured in triplicate on the same plate. The differences among parallels should be small (coefficient of variation was less than 10%) to guarantee the reproducibility of repeated measurements.

### Determination of enzyme activity

The frozen sample of the liver and longissimus dorsi (approximately 1.0 g) was homogenized in ice-cold saline solution (1:9, wt/vol), centrifuged at 3,000×*g* for 15 min at 4 °C, and stored at − 80 °C for further analysis. The activities of carnitine palmitoyltransferase 1 (CPT-1), lipoprotein lipase (LPL), hepatic lipase (HL), and malate dehydrogenase (MDH) in liver and longissimus dorsi were determined using commercial kits (Nanjing Jiancheng Bioengineering Institute, Nanjing, China) in accordance with the manufacturer’s instructions. The total protein concentration of liver and longissimus dorsi homogenates was measured by the Bradford brilliant blue method [24]. Each parameter was simultaneously determined in triplicate on the same plate. The differences among parallels should be small (coefficient of variation was less than 10%) to guarantee the reproducibility of repeated measurements.

### Detection of mRNA expression

Total RNA was isolated from the frozen colon, liver, and longissimus dorsi using Trizol reagent (TaKaRa) in accordance with the manufacturer’s instructions. The purity and concentration of the RNA were detected using a NanoDrop ND-2000 spectrophotometer (NanoDrop, Germany). The OD_260_:OD_280_ ratios ranging from 1.8 to 2.0 in all samples were considered as suitable for further measurement. The integrity of RNA was measured by agarose gel electrophoresis, and the 28S:18S ribosomal RNA band ratio was evaluated to be ≥1.8. RNA was reverse transcribed into cDNA by the PrimeScript^TM^ RT reagent kit (TaKaRa) following the manufacturer’s guidelines. Primers for the associated genes (Table S4) were designed via Primer 6 software (PREMIER Biosoft International, Palo Alto, CA, USA) and commercially synthesized by Sangon Biotech Ltd. (Shanghai, China). The quantitative real-time PCR was analyzed on an ABI Prism 7000 detection system in a two-step protocol with SYBR Green (Applied Biosystems, Foster City, CA, USA). Each reaction was contained in a volume of 5 μL SYBR Premix Ex Taq TM (2×), 1 μL cDNA, 0.4 μL of each forward and reverse primer, 0.2 μL ROX reference dye (50×), and 3 μL PCR-grade water. The PCR conditions were the initial denaturation at 95 °C for 30 s, followed by 40 cycles of denaturation at 95 °C for 10 s, then annealing at 60 °C for 25 s, and a 72 °C extension step for 5 min. The melting curve was formed following each quantitative real-time PCR determination to verify the specificity of the reactions. β-actin (housekeeping gene) was selected as the reference gene to normalize the mRNA expression of target genes. Gene abundance values of the replicate samples were computed by the 2^–ΔΔCT^ method [25]. The relative abundance of the target genes in the GF group was treat a to be 1.0. Each sample was determined in triplicate.

### Determination of protein levels

The antibodies against the β-actin, GPR43, p-AMPK, AMPK, CPT-1B, and ACC were brought from Abcam (Cambridge, MA, USA), Cell Signaling Technology (Davers, MA), and Santa Cruz Biotechnology Inc. (Santa Cruz, CA, USA), respectively. Protein levels for the β-actin, GPR43, p-AMPK, AMPK, CPT-1B, and ACC in the liver were measured by western blot analysis in accordance with the instructions described by Suryawan et al. [26].

### Ultrahigh-performance liquid chromatography equipped with quadrupole time of flight mass spectrometry (UHPLC-Q-TOF/MS) analysis

Serum samples were separated by the ultra-high-performance liquid chromatography (UHPLC) system (Agilent 1290, Agilent Technologies, Palo Alto, USA) incorporating a hydrophilic interaction liquid chromatography (HILIC) column (2.1 mm × 100 mm, 1.7 μm; Waters, Milford, MA, USA). The samples were analyzed using a triple time-of-flight (TOF) mass spectrometer (ESI/Triple TOF 5600; AB Sciex, Concord, Canada) equipped with an electrospray ionization source used in positive and negative ion modes. The pretreatment, extraction, and identification of serum samples were according to the procedure described by Hu et al. [27]. The raw data (whiff scan files) were converted into mzXML format using ProteoWizard [28] and were imported to the XCMS software for peak matching, retention time alignment, and peak area extraction [29]. Metabolite structure identification was performed by comparing the accuracy of m/z values (< 25 ppm), and MS/MS spectra were interpreted with an in-house database (Shanghai Applied Protein Technology Co. Ltd., China) established with authentic standards. For the XCMS data, the ion peaks that were missing values greater than 50% in the group were filtered and excluded and data were normalized to total peak intensity. Then, the pattern recognition was analyzed by SIMCA-P software (version 14.1, Umetrics, Umea, Sweden), where could performed to multivariate data measurement, including unsupervised principal component analysis (PCA), supervised partial least squares discriminant analysis (PLS-DA), and supervised orthogonal partial least squares discriminant analysis (OPLS-DA), which were carried out to uncover and extract the statistically significant metabolite variations. The PLS-DA and OPLS-DA models were validated based on multiple correlation coefficient (R^2^) and forecast ability according to the model (Q^2^) in cross-validation and permutation test by applying 200 iterations [30]. The R^2^ value in the permutated plot described how well the data fit the derived model, whereas the Q^2^ value described the predictive ability of the constructed model and was a measure of model quality [31]. Volcano Plot measurement synthesized *t*-test and Fold Change (FC) evaluation were to help identify potential metabolites. Metabolites with the highest variable importance in the projection (VIP) score are the most powerful group discriminators, VIP score > 1 are significant [32]. The significantly differential metabolites were ranked using the VIP score (> 1) based on the OPLS-DA model and *P* < 0.10. The instructions of metabolites identification and Kyoto Encyclopedia of Genes and Genomes (KEGG) pathway analysis were according to Wang et al.[30].

### Statistical analysis

Data were analyzed in SAS 9.2 (SAS Institute, Inc., Cary, NC, USA) and analyzed using Student’s *t*-test, and presented as means ± SEMs. The individual pig was the statistical unit. *P* < 0.05 was considered to be statistically significant, and tendency was declared with 0.05 < *P* < 0.10.

## Results

There were no differences in growth performance, nutrient digestibility, and relative organs weight between the GF and FA groups [33].

### Serum parameters

The impacts of oral administration of SCFAs on the serum parameters are shown in Table 1. Compared with the GF group, the FA group tended to had a higher adiponectin concentration in the serum (*P* < 0.10).

**Table 1:** Effects of exogenously introduced SCFAs on the serum parameters in GF pigs ^a^

Items	GF	FA	*P*-value
Adiponectin, μg/L	74.48 ± 1.62	80.63 ± 2.32	0.06
Glucagon, pg/mL	28.01 ± 1.52	30.06 ± 1.23	0.12
GLP-1, pmol/L	2.51 ± 0.06	2.57 ± 0.08	0.52
Insulin, mIU/L	10.20 ± 0.38	10.76 ± 0.52	0.40
Leptin, ng/L	1403.2 ± 38.30	1368.4 ± 45.03	0.57
TC, mmol/L	1.45 ± 0.10	1.59 ± 0.11	0.38
TG, mmol/L	0.30 ± 0.03	0.50 ± 0.12	0.15
HDL, mmol/L	0.73 ± 0.08	0.80 ± 0.06	0.51
LDL, mmol/L	0.77 ± 0.05	0.81 ± 0.06	0.65
Glucose, mmol/L	5.31 ± 0.31	5.33 ± 0.58	0.97

^a^ *GF* germ-free, *FA* short-chain fatty acids, *GLP-1* Glucagon like peptide 1, *TC* total cholesterol, *TG* triglyceride, *HDL-c* high-density lipoprotein-cholesterol, *LDL-c* low-density lipoprotein-cholesterol. Values are means ± SEMs, *n* = 6/group

### Activities of enzymes in the liver and longissimus dorsi

The activities of enzymes in the liver and longissimus dorsi are presented in Table 2. The activity of CPT-1 in the longissimus dorsi of the FA group was tended to be higher than that in the GF group (*P* < 0.10).

**Table 2:** Effects of exogenously introduced SCFAs on the activities associated with lipids metabolism in liver and longissimus dorsi of GF pigs ^a^

Items	GF	FA	*P*-value
Liver			
CPT-1, U/mg protein	192.40 ± 3.58	191.6 ± 7.27	0.92
LPL, U/mg protein	0.75 ± 0.02	0.73 ± 0.10	0.86
HL, U/mg protein	0.77 ± 0.11	0.78 ± 0.06	0.97
MDH, U/mg protein	43.56 ± 3.93	41.62 ± 2.48	0.68
Longissimus dorsi			
CPT-1, U/mg protein	197.70 ± 5.92	209.90 ± 1.91	0.08
LPL, U/mg protein	0.66 ± 0.26	0.34 ± 0.05	0.28
HL, U/mg protein	0.48 ± 0.05	0.39 ± 0.04	0.21
MDH, U/mg protein	4.08 ± 0.32	3.41 ± 0.16	0.10

^a^ *GF* germ-free, *FA* short-chain fatty acids, *CPT-1* carnitine palmitoyltransferase 1, *LPL* lipoprotein lipase, *HL* hepatic lipase, *MDH* malate dehydrogenase. Values are means ± SEMs, *n* = 6/group

### Relative mRNA expressions of lipid metabolism-related genes in liver and longissimus dorsi

As shown in Table 3, the mRNA expressions of *ANGPTL4* in the colon and *PGC-1α* in the liver of the FA group tended to upregulate (*P* < 0.10) compared with those in the GF group. Moreover, the mRNA abundances of *ACC*, *FAS*, and *SREBP-1C* in the liver of the FA group were lower than those in the GF group (*P* < 0.05). As presented in Table 4, oral administration of SCFAs decreased the mRNA expression of *CD36* (*P* < 0.05) and tended to downregulate the mRNA expression of *LPL* (*P* < 0.10) in the longissimus dorsi.

**Table 3:** Effects of exogenously introduced SCFAs on the mRNA abundances for key factors associated with lipid metabolism in colon and liver of GF pigs ^a^

Items	GF	FA	*P*-value
Colon			
*ANGPTL4*	1.00 ± 0.13	1.48 ± 0.17	0.06
*PPAR-γ*	1.00 ± 0.07	0.95 ± 0.14	0.75
Liver			
*ACC*	1.00 ± 0.17	0.63 ± 0.05	< 0.01
*FAS*	1.00 ± 0.16	0.56 ± 0.03	0.03
*CD36*	1.00 ± 0.25	0.60 ± 0.17	0.19
*LPL*	1.00 ± 0.11	0.69 ± 0.13	0.11
*SREBP-1C*	1.00 ± 0.09	0.72 ± 0.08	0.04
*PPKAA1*	1.00 ± 0.10	1.10 ± 0.09	0.47
*PPKAA2*	1.00 ± 0.13	0.99 ± 0.08	0.95
*CPT-1B*	1.00 ± 0.14	0.98 ± 0.11	0.93
*PGC-1α*	1.00 ± 0.16	2.02 ± 0.43	0.06
*PNPLA2*	1.00 ± 0.11	0.86 ± 0.15	0.47

^a^ *GF* germ-free, *FA* short-chain fatty acids, *ANGPTL4* angiopoietin-like 4, *PPAR-γ* peroxisome proliferator-activated receptor gamma, *ACC* acetyl-CoA carboxylase, *FAS* fatty acid synthase, *CD36* fatty acid transporter CD36, *LPL* lipoprotein lipase, *SREBP-1C* sterol regulatory element binding protein 1C, *PRKAA1* AMP activated alpha 1, *PRKAA2* AMP activated alpha 2, *CPT-1B* carnitine palmitoyltransferase 1 B, *PGC-1α* peroxisome proliferator-activated receptor gamma coactivator-1α, *PNPLA2* patatin-like phospholipase domain-containing protein 2. Values are means ± SEMs, *n* = 6/group

**Table 4:** Effects of exogenously introduced SCFAs on the mRNA abundances for key factors associated with lipid metabolism in longissimus dorsi of GF pigs ^a^

Items	GF	FA	*P*-value
*ACC*	1.00 ± 0.14	0.79 ± 0.03	0.19
*FAS*	1.00 ± 0.33	0.68 ± 0.06	0.37
*CD36*	1.00 ± 0.07	0.71 ± 0.06	0.02
*LPL*	1.00 ± 0.09	0.81 ± 0.04	0.08
*SREBP-1C*	1.00 ± 0.18	0.65 ± 0.06	0.11
*PPKAA1*	1.00 ± 0.08	0.91 ± 0.07	0.44
*PPKAA2*	1.00 ± 0.03	1.07 ± 0.06	0.39
*CPT-1B*	1.00 ± 0.15	1.14 ± 0.10	0.44
*PGC-1α*	1.00 ± 0.08	1.58 ± 0.39	0.19
*PNPLA2*	1.00 ± 0.16	0.97 ± 0.14	0.90

^a^ *GF* germ-free, *FA* short-chain fatty acids, *ACC* acetyl-CoA carboxylase, *FAS* fatty acid synthase, *CD36* fatty acid transporter CD36, *LPL* lipoprotein lipase, *SREBP-1C* sterol regulatory element binding protein 1C, *PRKAA1* AMP activated alpha 1, *PRKAA2* AMP activated alpha 2, *CPT-1B* carnitine palmitoyltransferase 1 B, *PGC-1α* peroxisome proliferator-activated receptor gamma coactivator-1α, *PNPLA2* patatin-like phospholipase domain-containing protein 2. Values are means ± SEMs, *n* = 6/group

### Relative mRNA expressions of glucose metabolism-related genes in liver and longissimus dorsi

As presented in Table 5, the oral administration of SCFAs upregulated the mRNA expressions of *GLU-2* and *GYS2* in the liver (*P* < 0.05). In addition, the mRNA abundances of genes related to glucose metabolism in the longissimus dorsi were no differences between the FA and GF groups (*P* > 0.10) (Table 6).

**Table 5:** Effects of exogenously introduced SCFAs on the mRNA abundances for key factors associated with glucose metabolism in liver of GF pigs ^a^

Items	GF	FA	*P*-value
*FOXO-1*	1.00 ± 0.05	1.09 ± 0.04	0.22
*INSR*	1.00 ± 0.06	0.81 ± 0.06	0.16
*INS1*	1.00 ± 0.10	1.02 ± 0.04	0.80
*PIK3*	1.00 ± 0.09	0.72 ± 0.02	0.12
*GLU-2*	1.00 ± 0.12	0.58 ± 0.08	< 0.01
*PCK1*	1.00 ± 0.05	1.08 ± 0.01	0.14
*GSK3*	1.00 ± 0.04	1.10 ± 0.05	0.17
*GYS2*	1.00 ± 0.01	1.67 ± 0.20	< 0.01

^a^ *GF* germ-free, *FA* short-chain fatty acids, *FOXO-1* foxo1 forkhead box O1, *INSR* insulin receptor, *IRS1* insulin receptor substrate 1, *PIK3* phosphatidylinositol 3-kinase catalytic subunit type 3, *GLU-2* glucose transporter 2, *PCK1* phosphoenolpyruvate carboxykinase 1, *GSK3* glycogen synthase kinase 3, *GYS2* glycogen synthase 2. Values are means ± SEMs, *n* = 6/group

**Table 6:** Effects of exogenously introduced SCFAs on the mRNA abundances for key factors associated with glucose metabolism in longissimus dorsi of GF pigs ^a^

Items	GF	FA	*P*-value
*FOXO-1*	1.00 ± 0.44	1.35 ± 0.34	0.20
*Sirt1*	1.00 ± 0.02	0.86 ± 0.03	0.11
*INSR*	1.00 ± 0.09	0.99 ± 0.21	0.97
*INS1*	1.00 ± 0.15	1.08 ± 0.09	0.64
*PIK3*	1.00 ± 0.09	0.96 ± 0.07	0.73
*GSK3*	1.00 ± 0.04	1.01 ± 0.08	0.91

^a^ *GF* germ-free, *FA* short-chain fatty acids, *FOX-1* foxo1 forkhead box O1, *Sirt1* silent information regulator 1, *INSR* insulin receptor, *IRS1* insulin receptor substrate 1, *PIK3* phosphatidylinositol 3-kinase catalytic subunit type 3, *GSK3* glycogen synthase kinase 3. Values are means ± SEMs, *n* = 6/group

### The protein level associated with lipid metabolism

**Fig. 1:**
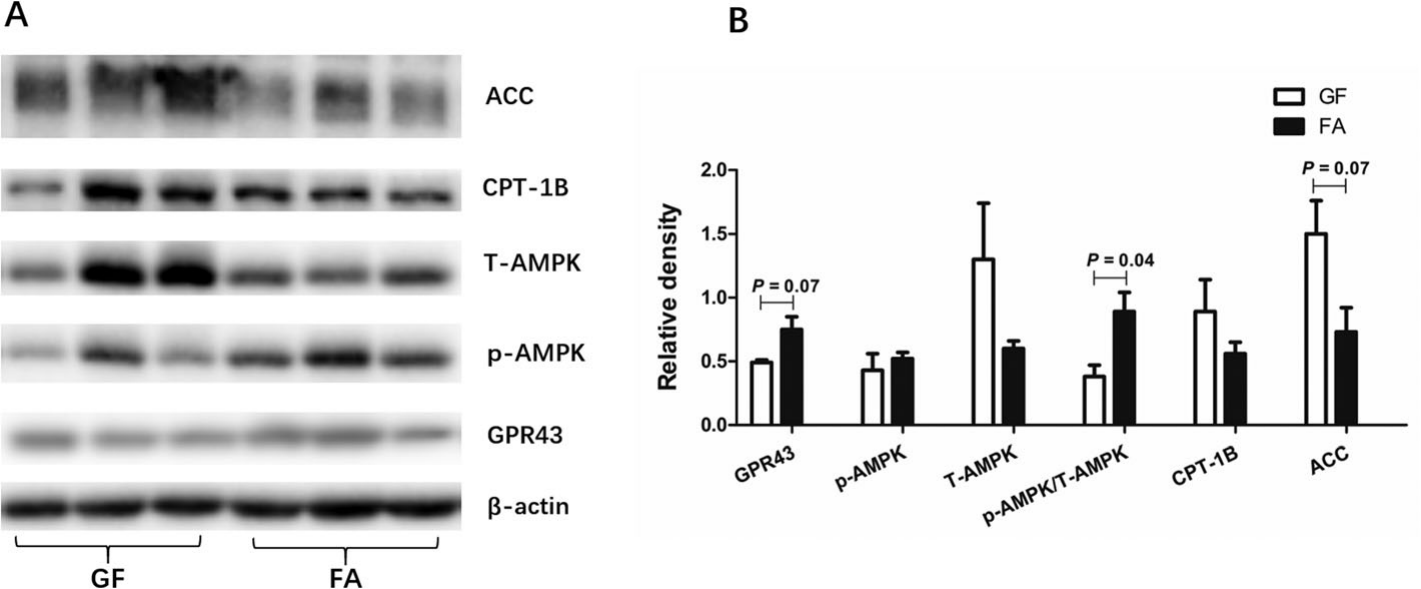
Effects of exogenously introduced SCFAs on the protein levels of GPR43, p-AMPK, AMPK, CPT-1B, and ACC in the liver of GF pigs. GF, germ-free; FA, short chain fatty acids; ACC, acetyl-CoA carboxylase; CPT-1B, carnitine palmitoyltransferase 1 B; p-AMPK, phosphorylated adenosine monophosphate-activated protein kinase; AMPK, adenosine monophosphate-activated protein kinase; GPR43, G-protein-coupled receptors 43

As presented in Fig. 1, oral administration of SCFAs tended to increase the protein level of GPR43 (*P* < 0.10) and tended to decrease the protein level of ACC (*P* < 0.10), while upregulated the p-AMPK/AMPK ratio (*P* < 0.05) in the liver.

### Metabolomic profile in the serum

**Fig. 2:**
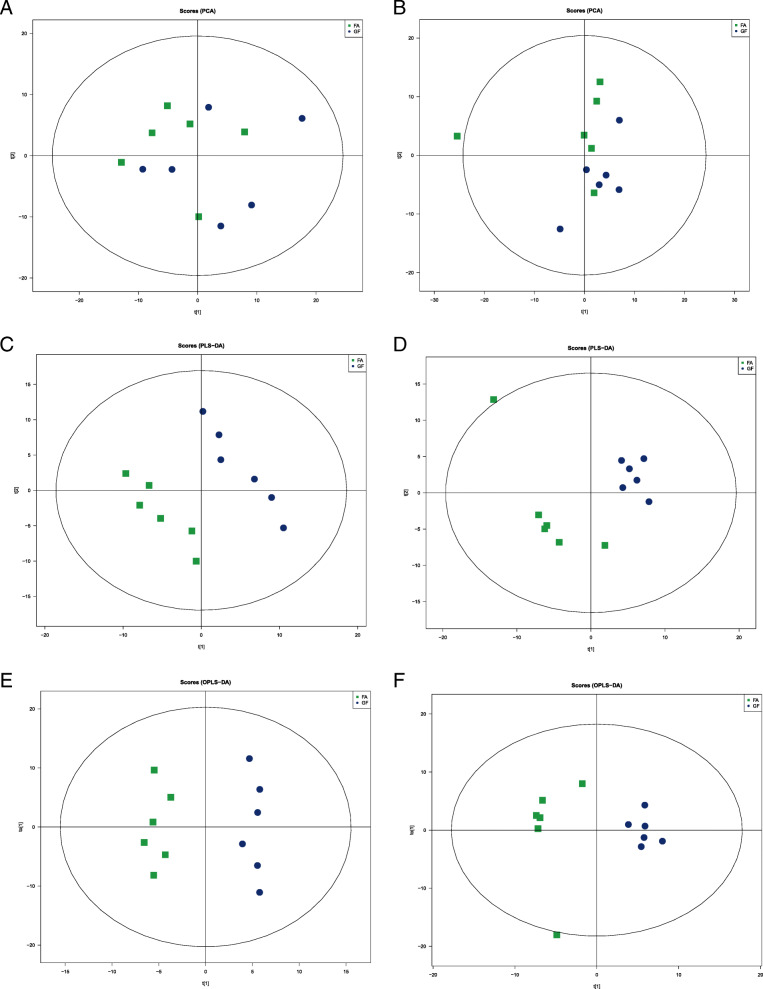
The PCA, PLS-DA, and OPLS-DA score plots comparing GF (blue rotundities) and FA (green squares) pigs in positive electrospray ionization mode (ESI^+^) and negative electrospray ionization mode (ESI^−^) metabolomics profiles of serum. Panels **a** (ESI^+^) and **b** (ESI^−^) are PCA score plots. Panels **c** (ESI^+^, R^2^X = 0.459, R^2^Y = 0.996, Q^2^ = 0.649) and **d** (ESI^−^, R^2^X = 0.514, R^2^Y = 0.999, Q^2^ = 0.818) are PLS-DA score plots. Panels **e** (ESI^+^, R^2^X = 0.317, R^2^Y = 0.976, Q^2^ = 0.009) and **f** (ESI^−^, R^2^X = 0.307, R^2^Y = 0.926, Q^2^ = 0.201) are OPLS-DA score plots. GF, germ-free; FA, short chain fatty acids; PCA, principal component analysis; PLS-DA, Partial least squares discriminant; OPLS-DA, orthogonal partial least-squares discriminant

**Fig. 3:**
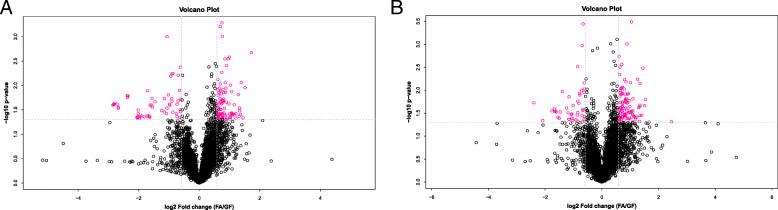
Volcano plots showing the distribution of all metabolites based on their fold-change values (X-axis, on a logarithmic scale), *P*-value (Y-axis, on a logarithmic scale). Panel **a** is ESI^+^, Panel **b** is ESI^−^, respectively (FC > 1.50 and *P*-value < 0.05). GF, germ-free; FA, short chain fatty acids

**Fig. 4:**
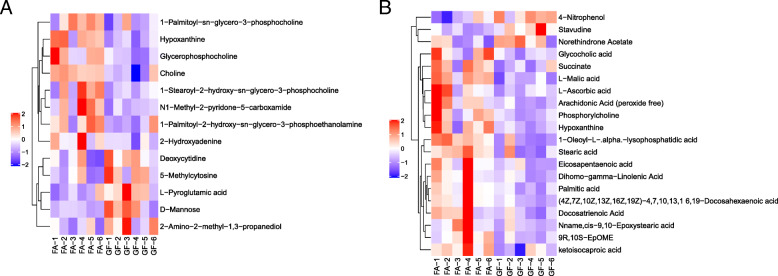
Hierarchical clustering heat map of significantly differential metabolites from serum of pigs in the ESI^+^ (**a**) and ESI^−^ (**b**). Metabolites peak area were Z score transformed. Warm color and cold color indicate increased and decreased expression of the metabolites, respectively. GF, germ-free; FA, short chain fatty acids

**Fig. 5:**
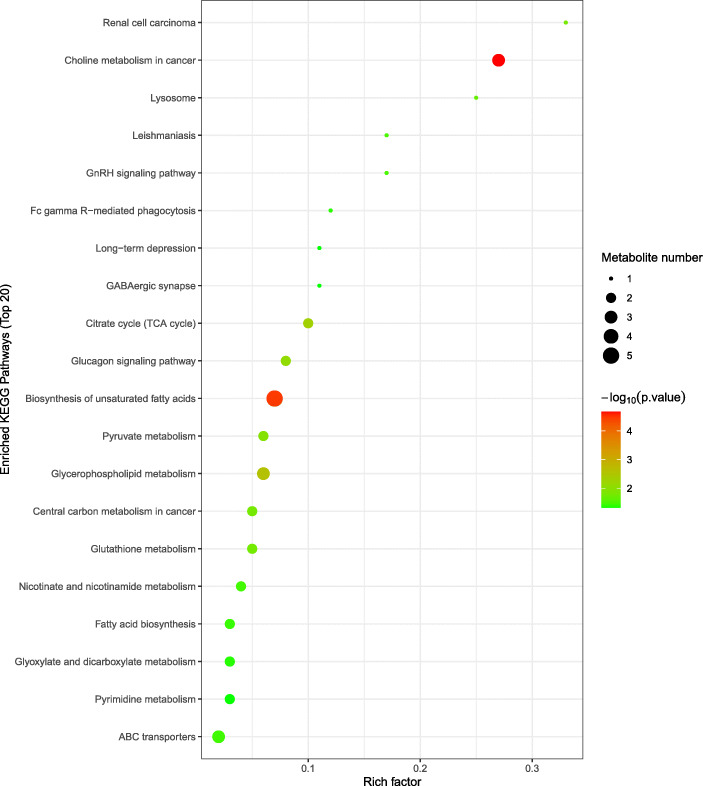
Topology analysis of metabolic pathways identified between GF and FA groups. The X-axis represents the rich factor, and the Y-axis represents the pathway. Larger sizes and darker colors represent greater pathway enrichment and higher pathway impact values, respectively. GF, germ-free; FA, short chain fatty acids

To further evaluate the differences in metabolic profiles related to lipid and glucose metabolism between GF and FA groups, UHPLC-QTOF-MS was used to identify the differential metabolites. Serum samples from GF and FA pigs were measured in both positive and negative ionization modes. The PCA, PLS-DA, and OPLS-DA were performed to visualize the LC-MS dataset and exhibit the differences and similarities among the samples. No marked difference between GF and FA groups in PCA analysis (Fig. 2a, b). To further dissect the difference between GF and FA groups, the PLS-DA and OPLS-DA analyses were employed. The PLS-DA (Fig. 2c, d) and OPLS-DA (Fig. 2e, f) score plots show separation between the GF and FA groups in both positive and negative modes. As presented in Fig. 3, the differential metabolites change between GF and FA groups were exhibited by univariate measurement. The red dots exhibit metabolites that differed between GF and FA groups undertaking FC > 1.5 and *P*-value < 0.05. To assess which compounds were responsible for the differences between the two groups, the parameters of VIP > 1.0 and adjusted *P* < 0.10 were used as key lineages for separating the serum compounds between GF and FA groups (Fig. 4 and Table 7). In total, 33 compounds with a VIP > 1.0 and adjusted *P* < 0.10 were identified. Among these, 17 metabolites (choline, hypoxanthine, glycerophosphocholine, N1-methyl-2-pyridone-5-carboxamide, *L*-malic acid, 1-oleoyl-*L*-alpha-lysophosphatidic acid, arachidonic acid, stearic acid, ketoisocaproic acid, hypoxanthine, 9*R*-10*S*-EpOME, phosphorylcholine, succinate, docosahexaenoic acid, docosatrienoic acid, and palmitic acid) were enriched (*P* < 0.05) and four metabolites (*D*-mannose, *L*-pyroglutamic acid, 4-nitrophenol, and stavudine) were reduced (*P* < 0.05) in the FA group compared with those in the GF group. Overall, these results suggested that oral administration of SCFAs markedly increased the lipids related compounds (arachidonic acid, stearic acid, docosahexaenoic acid, palmitic acid, glycerophosphocholine), indicating that exogenous SCFAs had a strong impact on the lipid metabolism in pigs. To further understand the physiological difference induced by oral administration of SCFAs, the KEGG pathway database was used to identifying associated metabolic pathways of 33 metabolites detected in serum. According to Fig. 5, these compounds were involved in several biochemical pathways, and the biosynthesis of unsaturated fatty acids pathway was most significantly affected (*P* < 0.05) by exogenous SCFAs.

**Table 7:** Altered metabolites in the serum between FA and GF groups ^a^

Adduct	Metabolites	Metabolic pathway	M-to-Z	Rt, s	VIP	FC	*P*-value
M+	Choline	Glycerophospholipid metabolism	104.11	559.22	3.47	1.25	0.0042
(M + H)+	Hypoxanthine	Purine metabolism	137.05	349.60	6.37	1.33	0.0058
M+	Glycerophosphocholine	Glycerophospholipid metabolism	258.11	781.81	8.80	1.53	0.0124
(M + H)+	N1-Methyl-2-pyridone-5-carboxamide	Nicotinate and nicotinamide metabolism	153.07	127.37	1.36	1.32	0.0212
(M + Na)+	*D*-Mannose	Lysosome	203.05	574.98	1.70	0.65	0.0314
(M + H)+	L-Pyroglutamic acid	Glutathione metabolism	130.05	751.76	1.85	0.65	0.0344
(M + Na)+	1-Palmitoyl-sn-glycero-3-phosphocholine	―	518.32	386.60	1.49	1.24	0.0575
(M-H + 2Na)+	1-Stearoyl-2-hydroxy-sn-glycero-3-phosphocholine	―	568.34	367.11	4.30	1.74	0.0699
(M + H)+	5-Methylcytosine	Pyrimidine metabolism	126.07	403.47	1.05	0.66	0.0741
(M + H)+	1-Palmitoyl-2-hydroxy-sn-glycero-3-phosphoethanolamine	―	454.29	396.52	1.27	1.57	0.0790
(M + H-2H_2_O)+	2-Amino-2-methyl-1,3-propanediol	―	70.06	541.31	1.15	0.78	0.0801
(M + H)+	2-Hydroxyadenine	Purine metabolism	152.06	457.91	1.50	1.32	0.0813
(M + H)+	Deoxycytidine	Pyrimidine metabolism	228.10	421.57	1.02	0.74	0.0881
(M-H)-	*L*-Malic acid	Renal cell carcinoma	133.01	727.91	1.80	1.85	0.0009
(M + Na-2H)-	1-Oleoyl-*L*-alpha-lysophosphatidic acid	―	457.23	438.26	1.67	1.63	0.0027
(M-H)-	4-Nitrophenol	―	138.02	59.08	1.05	0.82	0.0167
(M-H)-	Arachidonic acid	Biosynthesis of unsaturated fatty acids	303.23	67.07	16.18	1.77	0.0170
(M-H)-	Stearic acid	Biosynthesis of unsaturated fatty acids	283.26	349.68	1.11	1.58	0.0183
(M-H)-	Ketoisocaproic acid	Valine, leucine and isoleucine degradation	129.06	94.17	5.75	1.20	0.0218
(M + Na-2H)-	Stavudine	―	245.05	539.43	1.16	0.30	0.0281
(M-H)-	Hypoxanthine	Purine metabolism	135.03	298.92	7.13	1.25	0.0335
(M-H)-	9*R*,10*S*-EpOME	―	295.23	75.32	1.46	1.68	0.0373
(M + CH_3_COO)-	Phosphorylcholine	Glycerophospholipid metabolism	242.08	721.65	1.32	2.28	0.0387
(M-H)-	Succinate	Citrate cycle (TCA cycle)	117.02	697.09	1.81	1.30	0.0409
(M-H)-	(4*Z*,7*Z*,10*Z*,13*Z*,16*Z*,19*Z*)-4,7,10,13,1 6,19-Docosahexaenoic acid	Biosynthesis of unsaturated fatty acids	327.23	67.13	8.88	1.87	0.0413
(M-H)-	Docosatrienoic acid	―	333.28	64.84	1.15	1.44	0.0459
(M-H)-	Palmitic acid	Biosynthesis of unsaturated fatty acids	255.23	89.61	7.25	2.51	0.0478
(M-H)-	Eicosapentaenoic acid	Biosynthesis of unsaturated fatty acids	301.22	68.36	4.80	1.90	0.0505
(M-H)-	Dihomo-gamma-linolenic acid	―	305.25	66.69	4.36	1.67	0.0543
(M-H)-	*L*-Ascorbic acid	Glutathione metabolism	175.02	660.84	2.19	2.01	0.0617
(M-H)-	Norethindrone acetate	―	339.20	1152.64	1.82	0.64	0.0852
(M-H)-	Glycocholic acid	Bile secretion	464.31	322.78	1.41	1.43	0.0884
(M-H)-	Nnamecis-9,10-epoxystearic acid	―	297.24	75.71	1.42	1.82	0.0895

^a^ *GF* germ-free, *FA* short chain fatty acids, *M-to-Z* mass-to-charge ratio, *Rt* retention time; the VIP value was obtained from OPLS-DA model with a threshold of 1.0; *FC* foldchange, was calculated by dividing the mean intensity of FA group pig’s serum metabolites by the mean intensity of GF group pig’s serum metabolites; the significance *P*-value was obtained from Student’s test with a threshold of 0.10

## Discussion

As is known to us, increasing dietary fiber intake contributes greatly to body weight and glucose tolerance [34]. Notably, when introduced with SCFAs, the SCFAs concentrations in the circulation or gut were similar to those observed in a higher fiber diet [35]. Indeed, it has been reported that exogenous introduction of SCFAs attenuated the body fat deposition in both mice, pigs, and humans [7, 10, 11]. Although SCFAs can prevent fat accumulation, while the underlying mechanisms are still not fully understood. SCFAs can be produced naturally by host metabolic pathways particularly in the liver, but the major site of SCFAs production is the colon which requires the presence of specific bacteria [36]. Moreover, the numbers and diversity of microbiota are positively associated with SCFAs concentrations [19], and several specific microbes species were closely related to host lipid metabolism [16, 17]. However, whether the SCFAs regulate lipid and glucose metabolism independent of the gut microbiota are largely unknown. Therefore, this study was conducted to explore the effects of exogenous introduction of SCFAs on lipid and glucose metabolism in a GF pig model and to further dissect the underlying mechanisms of exogenously introduced SCFAs on the host’s health. To determine the concentration and dose of the SCFAs mixture used in the present experiment, we conducted a preliminary experiment on the conventional Bama piglets (*n* = 12). In the preliminary trial, when pigs fed with 1.25 S_0_ (acetic, propionic, and butyric acids, 75, 25, and 19 mmol/L, respectively) and 25 mL/kg or 1.0 S_0_ (acetic, propionic, and butyric acids, 60, 20, and 15 mmol/L, respectively) and 25 mL/kg SCFAs mixture led to diarrhea and death. Meanwhile, the feed intake was not decreased and without diarrhea after 7 d when pigs fed with 0.75 S_0_ (acetic, propionic, and butyric acids, 45, 15, and 11 mmol/L, respectively) and 25 mL/kg SCFAs mixture. However, when pigs fed with 0.75 S_0_ and 35 mL/kg SCFAs also led to diarrhea and reduced feed intake. Taken together, we determined the mixture concentrations of acetic, propionic, and butyric acids at 45, 15, and 11 mmol/L, respectively, and a dose of 25 mL/kg in the present study.

Acting in peripheral tissues, adiponectin could regulate lipid metabolism and promote energy expenditure [37]. Serum adiponectin concentration was reduced in individuals with obesity and obesity-related diseases [38]. In the present study, oral administration of SCFAs tended to increase the concentration of adiponectin in serum. CPT-1 is the rate-limiting enzyme that determines fatty acids oxidation [39]. *ANGPTL4* is a valid inhibitor of lipoprotein lipase to regulate cellular uptake of triglycerides and promote fatty acids oxidation [40, 41]. In our study, oral administration of SCFAs tended to increase the activity of CPT-1 in longissimus dorsi and the mRNA abundance of *ANGPTL4* in colon. Meanwhile, we found the mRNA expressions of *FAS*, *ACC*, and *SREBP-1C* in liver of the FA group markedly downregulated compared with those in the GF group. Consistently, previous studies reported similar results in liver, longissimus dorsi, and adipose tissues of conventional pigs [9, 10, 42]. Notably, *FAS* is the pivotal enzyme that catalyzes fatty acids synthesis [43]. *ACC* modulates fatty acids metabolism, and its product (e.g. malonyl-CoA) serves as a building block for *de novo* fatty acids synthesis [44]. The *SREBPs* increases the transcription of genes that encode the enzymes of fatty acids biosynthesis and cholesterol uptake [45]. In addition, the current study observed the mRNA abundance of *CD36* was apparently decreased, and *LPL* tended to be reduced in longissimus dorsi of the FA group. *LPL* catalyzes the hydrolysis of triglycerides residing in chylomicrons and providing free fatty acids for tissue utilization [39]. *CD36*, the fatty acids translocase, regulates the uptake of long-chain fatty acids into cells [46], and elevated expression of *CD36* in various tissues resulted in lipid overload and lipotoxicity [47]. Noteworthy, the *PGC-1α* was measured as a vital regulator of fatty acids metabolism [48], and increasing the *PGC-1α* expression in liver was a negative association with body fat [49]. In the present study, oral administration of SCFAs tended to upregulate the mRNA expression of *PGC-1α* in liver, in agreement with the previous studies in conventional pigs and mice [7, 10]. Besides, SCFAs have been demonstrated to enhance the rates of oxygen consumption, and to increase both fat oxidation and adaptive thermogenesis in rodents [7, 50]. Collectively, these demonstrated that exogenous SCFAs may decrease the lipid deposition by downregulating the mRNA expressions of genes related to fatty acids synthesis and enhancing energy expenditure in the liver and longissimus dorsi of GF pigs.

The liver also plays a central role in regulating blood glucose homeostasis by uptake of glucose in the postprandial state and conversion to glycogen and triglyceride, and via the production of glucose in the postabsorptive state through glycogenolysis and gluconeogenesis [51, 52]. The rate-limiting enzyme for glycogen synthesis is glycogen synthase (GS), in mammals, there are two GS isoforms: muscle GS (encoded by *GYS1*) is abundantly expressed in skeletal and cardiac muscles, and the liver-restricted isoform (encoded by *GYS2*) [53]. Previous work indicated that mice lacked *GYS2* had a severe decrease in their ability to store glycogen in hepatocytes [53]. It is well exhibited that insulin resistance and hepatic steatosis lead to compromised glycogen synthesis [54]. On the contrary, an increase in liver glycogen synthesis directly associated with improved glucose tolerance [55]. In the present study, oral administration of SCFAs significantly increased the mRNA expression of *GYS2* in the liver. Similarly, it has been shown that SCFAs supplementation reduced adiposity and improved glucose homeostasis compared to the control group [56]. The *GLUT-2* transports glucose in the liver to pass the membrane in a bi-directional way for glycolysis and gluconeogenesis and was identified as a major contributor to glucose and fructose homeostasis in the liver [57]. The increase in the expression of *GLUT-2* in liver may be associated with insulin resistance and type-2 diabetes mellitus [58]. In the current study, we found that oral administration of SCFAs markedly downregulated the mRNA abundance of *GLU-2* in liver. These suggested that exogenous SCFAs may improve glucose control in the liver of GF pigs.

Although previous scientists had done much work, the underlying mechanisms of SCFAs on lipid and glucose metabolism are still not fully understood. G-protein-coupled receptors (GPRs), GPR41 and GPR43 have been demonstrated to be indispensable for a range of SCFA-mediated effects [59, 60]. SCFAs have been shown to promote energy consumption and fat combustion by activating the GPRs [61]. Meanwhile, it has been indicated that GPR43 knockout mice exhibited a reduction in energy expenditure, while overexpression of GPR43 exhibited an increase in energy expenditure [62]. Moreover, the effects of SCFAs involving improvement of insulin response are also regulated by GPR43, which induces enhanced glucose control [63]. In our study, we observed the protein expression level of GPR43 in liver of the FA group tended to be upregulated compared with that in the GF group. In addition to the SCFAs-GPRs pathway being involved in the regulation of lipid and glucose metabolism, adenosine monophosphate-activated protein kinase (AMPK) also plays an important role in this regulation. Accumulating evidence demonstrated that SCFAs could increase AMPK activity in the liver and muscle [7, 64]. In the present study, the ratio of p-AMPK/AMPK was significantly increased in the FA group. Additionally, SCFAs were found to mediate liver lipid and glucose homeostasis via activating the PPAR-dependent AMPK-ACC pathway, which regulated the effects of SCFAs on gluconeogenesis and lipogenesis [8]. Of note, the present study observed the protein expression level of ACC in liver of the FA group tended to be higher than that in the GF group. These findings indicated that exogenous SCFAs may decrease fat storage and improve glucose control by binding to the GPR43 and activating the AMPK-ACC pathway in GF pigs.

To further understand the underlying mechanisms of SCFAs on lipid and glucose metabolism, metabolomics analysis was introduced in the present study. Metabolomics is a pyramidally used tool for exhaustive research of all metabolites comprised in an organism [65], which offers a novel strategy to identify the potential markers and to explore the molecular mechanisms and metabolic pathways response to specific nutritional interventions. Importantly, the serum can be regarded as a metabolic fingerprint that provides visual results of the metabolic differences and reveals the changes in metabolic pathways under various physiological or nutritional conditions [66]. In the present study, PLS-DA and OPLS-DA analyses demonstrated a clear separation of serum metabolites due to oral administration of SCFAs, suggesting marked differences in the metabolic profiles. Indeed, several fatty acids, such as stearic acid, arachidonic acid, docosahexaenoic acid, and palmitic acid in the FA group apparently increased compared with those in the GF group. Increases in serum fatty acids levels implied that lipid metabolisms have been altered. Taken KEGG pathway analysis, we observed these fatty acids (stearic acid, arachidonic acid, docosahexaenoic acid, palmitic acid) were involved in the biosynthesis of unsaturated fatty acids pathway. Of note, oral administration of SCFAs has the most significant impact on this metabolic pathway. Intake of unsaturated fatty acids, which consist of monounsaturated fatty acids and polyunsaturated fatty acids, has been associated with favorable cardiac diastolic function and body composition in obese patients [67]. Moreover, increasing unsaturated fatty acids in the diet also prevented weight gain and cardiac dysfunction in a mouse model [68]. Consequently, these suggested that exogenous introduction of SCFAs may alleviate the lipid deposition via activating the metabolic pathway of biosynthesis of unsaturated fatty acids in GF pigs.

## Conclusions

In summary, this study demonstrated that SCFAs may attenuate fat deposition and to some extent improve glucose control in the liver and longissimus dorsi, which occur independently of the gut microbiota. The possible mechanisms of exogenous SCFAs on lipid reduction and glucose tolerance improvement may be via binding to the GPR43 and activating the AMPK-ACC pathway, and stimulating the metabolic pathway of biosynthesis of unsaturated fatty acids in GF pigs. The current work further suggested the importance of the presence of gut microbes and provided novel evidence that exogenous introduction of SCFAs may be a possible therapeutic strategy to prevent metabolic disorders and to counteract the gut microbiota deficiency or imbalance.

## Supplementary Information

**Additional file 1 **: **Table S1.** Ingredient composition of the milk powder (as-fed basis). **Table S2.** Ingredient composition of the basal diet (as-fed basis). **Table S3.** Infusion volume of sterile saline or SCFAs mixture for each pig per day. **Table S4.** Primer sequences used for real-time quantitative PCR.

## Data Availability

The data were exhibited in the main manuscript and supplemental materials.

## Abbreviations

ACC: Acetyl-CoA carboxylase
AMPK: Adenosine monophosphate-activated protein kinase
ANGPTL4: Angiopoietin-like 4
CD36: Fatty acid transporter CD36
CPT-1B: Carnitine palmitoyltransferase 1 B
FAS: Fatty acid synthase
FOXO-1: Foxo1 forkhead box O1
G6PC: Glucose-6-phosphatase
GF: Germ-free
GLU-2: Glucose transporter 2
GPR43: G-protein-coupled receptors 43
GSK3: Glycogen synthase kinase 3
GYS2: Glycogen synthase 2
HDL-c: High density lipoprotein-cholesterol
HL: Hepatic lipase
INSR: Insulin receptor
IRS1: Insulin receptor substrate 1
LDL-c: Low density lipoprotein-cholesterol
LPL: Lipoprotein lipase
MDH: Malate dehydrogenase
OPLS-DA: Orthogonal partial least squares discriminant analysis
p-AMPK: Phosphorylated adenosine monophosphate-activated protein kinase
PCK1: Phosphoenolpyruvate carboxykinase 1
PIK3: Phosphatidylinositol 3-kinase catalytic subunit type 3
PGC-1α: Peroxisome proliferator-activated receptor gamma coactivator-1α
PLS-DA: Partial least squares discriminant analysis
PNPLA2: Adipose triglyceride lipase
PPAR-γ: Peroxisome proliferator-activated receptor gamma
PRKAA1: AMP activated alpha 1
PRKAA2: AMP activated alpha 2
Sirt1: Silent information regulator 1
SREBP-1C: Sterol regulatory element binding protein 1C
TC: Total cholesterol
TG: Triglyceride
UHPLC-Q-TOF/MS: Ultrahigh-performance liquid chromatography equipped with quadrupole time of-flight mass spectrometry
VIP: Variable importance in the projection
