# Supplementary material for: Short-chain fatty acids can improve lipid and glucose metabolism independently of the pig gut microbiota
Source: J Anim Sci Biotechnol. 2021 May 6;12:61. doi: 10.1186/s40104-021-00581-3 (PMC8101156; doi:10.1186/s40104-021-00581-3)
Supplement: Supplementary file 1 — Additional file 1 : Table S1. Ingredient composition of the milk powder (as-fed basis). Table S2. Ingredient composition of the basal diet (as-fed basis). Table S3. Infusion volume of sterile saline or SCFAs mixture for each pig per day. Table S4. Primer sequences used for real-time quantitative PCR. [file 40104_2021_581_MOESM1_ESM.docx]

**Table S1** Ingredient composition of the milk powder (as-fed basis)

| Ingredients, % | % | Calculated nutrient levels ^c^ |  |
| --- | --- | --- | --- |
| Whole milk powder | 60.00 | CP, % | 28.65 |
| Whey protein concentrate | 25.00 | Digestible energy, kcal/kg | 4,700 |
| Casein | 5.80 | SID-Lysine, % | 2.29 |
| Coconut oil | 3.00 | SID-Methionine, % | 0.89 |
| Soy lecithin | 0.05 | SID-Threonine, % | 1.46 |
| Glucose | 4.00 | SID-Tryptophan, % | 0.61 |
| Sweeteners | 0.10 | Calcium, % | 0.99 |
| Choline chloride | 0.10 | Available phosphorus, % | 0.62 |
| *L*-Lysine-HCl | 0.10 |  |  |
| *DL*-Methionine | 0.25 |  |  |
| *L*-Threonine | 0.25 |  |  |
| *L*-Tryptophan | 0.10 |  |  |
| *L*-Arginine | 0.20 |  |  |
| *L*-Glutamine | 0.50 |  |  |
| Mineral premix ^a^ | 0.35 |  |  |
| Vitamin premix ^b^ | 0.20 |  |  |
| Total | 100.00 |  |  |

^a^ supplemented following per kilogram of diet: Fe, 100 mg as FeSO_4_; Cu, 20 mg as CuSO_4_·5H_2_O, Zn, 100 mg as ZnSO_4_, Mn, 60 mg as MnSO_4_, I, 0.3 mg as KI, and Se, 0.3 mg as Na_2_SeO_3_.

^b^ Provided the following per kilogram of diet: vitamin A, 12,000 IU; vitamin D_3_ 3,000 IU; vitamin E, 30 IU; vitamin B_1_, 2.0 mg; vitamin B_2_, 8.0 mg; vitamin B_12_, 0.04 mg; vitamin B_6_, 3.0 mg; vitamin K, 50 mg; calcium pantotenate, 15 mg; nicotinic acid, 20 mg; biotin, 0.15 mg; folic acid, 0.8 mg;

^c^ Values for standardized ileal concentrations of amino acids were estimated using standardized ileal digestible (SID) coefficients provided by NRC (2012), for amino acids and digestive energy data also obtained from it.

**Table S2** Ingredient composition of the basal diet (as-fed basis)

| Ingredients, % | % | Calculated nutrient level ^b^ |  |
| --- | --- | --- | --- |
| Corn | 14.10 | CP, % | 19.00 |
| Puffed corn | 10.00 | Digestive energy, Kcal/kg | 3,622 |
| Soybean meal | 13.65 | SID-Lysine , % | 1.23 |
| Puffing of soybean | 7.00 | SID-Methionine, % | 0.36 |
| Soy protein concentrate | 5.00 | SID-Threonine, % | 0.73 |
| Whey powder | 5.00 | SID-Tryptophan, % | 0.20 |
| Fish meal | 3.00 | Calcium, % | 0.70 |
| Plasma protein powder | 3.00 | Available phosphorus, % | 0.45 |
| Glucose | 2.00 |  |  |
| Soybean oil | 1.40 |  |  |
| Maize starch | 29.70 |  |  |
| Dietary fiber | 3.00 |  |  |
| Limestone | 0.45 |  |  |
| Dicalcium phosphate | 1.25 |  |  |
| *L*-Lysine-HCl | 0.29 |  |  |
| *DL*-Methionine | 0.12 |  |  |
| *L*-Threonine | 0.13 |  |  |
| *L*-Tryptophan | 0.01 |  |  |
| Mineral-vitamin premix ^a^ | 0.50 |  |  |
| NaCl | 0.25 |  |  |
| Choline chloride | 0.15 |  |  |
| Total | 100.00 |  |  |

^a^ Provided the following per kilogram of diet: vitamin A, 8,000 IU; vitamin D_3_ 2,000 IU; vitamin E, 20 IU; vitamin B_1_, 1.5 mg; vitamin B_2_, 5.6 mg; vitamin B_12_, 0.02 mg; vitamin B_6_, 1.5 mg; vitamin K, 32 mg; calcium pantotenate, 10 mg; nicotinic acid, 15 mg; biotin, 0.1 mg; folic acid, 0.6 mg; Fe, 100 mg as FeSO_4_; Cu, 20 mg as CuSO_4_·5H_2_O, Zn, 100 mg as ZnSO_4_, Mn, 60 mg as MnSO_4_, I, 0.3 mg as KI, and Se, 0.3 mg as Na_2_SeO_3_.

^b^ Values for standardized ileal concentrations of amino acids were estimated using standardized ileal digestible (SID) coefficients provided by NRC (2012), for amino acids and digestive energy data also obtained from it.

Table S3 Infusion volume of sterile saline or SCFAs mixture for each pig per day ^a^

| ID of piglet | BW_0_, kg | d 1-5 | | d 6-10 | | d 11-15 | | d 16-21 | |
| --- | --- | --- | --- | --- | --- | --- | --- | --- | --- |
|  |  | BW_1_, kg | Dose_1_, mL | BW_2_, kg | Dose_2_, mL | BW_3_, kg | Dose_3_, mL | BW_4_, kg | Dose_4_, mL |
| GF-1 | 2.60 | 3.05 | 76.25 | 3.5 | 87.5 | 3.95 | 98.75 | 4.4 | 110 |
| GF-2 | 2.65 | 3.10 | 77.5 | 3.55 | 88.75 | 4 | 100 | 4.45 | 111.25 |
| GF-3 | 2.25 | 2.70 | 67.5 | 3.15 | 78.75 | 3.6 | 90 | 4.05 | 101.25 |
| GF-4 | 2.75 | 3.2 | 80 | 3.65 | 91.25 | 4.1 | 102.5 | 4.55 | 113.75 |
| GF-5 | 2.55 | 3.00 | 75 | 3.45 | 86.25 | 3.9 | 97.5 | 4.35 | 108.75 |
| GF-6 | 2.45 | 2.9 | 72.5 | 3.35 | 83.75 | 3.8 | 95 | 4.25 | 106.25 |
| SCFA-1 | 2.60 | 3.05 | 76.25 | 3.5 | 87.5 | 3.95 | 98.75 | 4.4 | 110 |
| SCFA-2 | 2.75 | 3.2 | 80 | 3.65 | 91.25 | 4.1 | 102.5 | 4.55 | 113.75 |
| SCFA-3 | 2.70 | 3.15 | 78.75 | 3.6 | 90 | 4.05 | 101.25 | 4.5 | 112.5 |
| SCFA-4 | 2.65 | 3.1 | 77.5 | 3.55 | 88.75 | 4 | 100 | 4.45 | 111.25 |
| SCFA-5 | 2.70 | 3.15 | 78.75 | 3.6 | 90 | 4.05 | 101.25 | 4.5 | 112.5 |
| SCFA-6 | 2.85 | 3.30 | 82.5 | 3.75 | 93.75 | 4.2 | 105 | 4.65 | 116.25 |

^a^ BW_0_, initial weight, BW_1_ = BW_0_×5×0.09 (according to the average daily gain of conventional Bama pigs, we estimated that the average daily gain of GF Bama pigs was 0.09 kg, and estimate every five days). BW_2_ = BW_1_×5×0.09, BW_3_ = BW_2_×5×0.09, BW_4_ = BW_3_×5×0.09. Dose_1_ = BW_1_×25 mL/kg, Dose_2_ = BW_2_×25 mL/kg, Dose_3_ = BW_3_×25 mL/kg, Dose_4_ = BW_4_×25 mL/kg.

**Table S4** Primer sequences used for real-time quantitative PCR

| Target gene | Forward primer (5’→3’) | Reverse primer (5’→3’) | Product length, bp | Accession number |
| --- | --- | --- | --- | --- |
| *β*-actin | TCTGGCACCACACCTTCT | TGATCTGGGTCATCTTCTCAC | 114 | XM_021086047.1 |
| *ANGPTL4* | TGGTGGTTGGTGGTTTGGCAC | TCGGCTACTGTGGGCTGGAT | 171 | NM_001038644.1 |
| *PPAR-γ* | TCCAGCATTTCCACTCCACAC | GGGACACAGGCTCCACTTTG | 127 | NM_214379.1 |
| *ACC* | TGTCCACTCAAGCATACCTCCCA | GCTACCATGCCAATCTCATTTCCTCC | 136 | NM_001114269 |
| *FAS* | GCCGAGTACAGCGTCAACAACC | TGGTCCTTCTTCATCAGCGGGAT | 172 | NM_001099930 |
| *CD36* | CTGTGGACTCATTGCTGGTGCTG | AAAACTGTCTGTAAACTTCCGTGCCT | 179 | NM_001044622 |
| *LPL* | AACGTCATTGTGGTGGACTGGCT | TCCAAGGCTGTATCCCAGGAGGTG | 177 | NM_214286.1 |
| *SREBP-1C* | TCCGTGAACACCTCTTGGAGCA | GCTGGAGGCAATGGAGAAGCTG | 173 | NM_214157 |
| *PRKAA1* | TCAGGGACTGCTACTCCACAGAGA | AAGAGTCAAGTGAGGTTACAGATGAGGT | 136 | NM_001167633 |
| *PRKAA2* | CCAGTGAGTTCTACCTCGCCTCT | TGGACATCTTGCTTTAGGGCTGTCT | 140 | NM_214266 |
| *CPT-1B* | AGTCATGGTGGGCGACTAACTATGTG | ATCATGGCGTGGACAGCGTTC | 169 | NM_001007191 |
| *PNPLA2* | CCTGCCTCTCTACGAACTCAAGAGC | AGGCTGAACTGGATGCTGGTGT | 132 | NM_001098605 |
| *PGC-1α* | CACCA GCCAA CACTC AGCTA | GAGGT GCACT TGTCT CTGCT | 111 | NM_213963.1 |
| *FOX-1* | GTCTT CACCA GGCAC CATCA | TTTTG GTAGT TGGGG CTGGG | 93 | NM_214014.2 |
| *Sirt1* | TGACT GTGAAGCTGTACGAG GAG | TGGCT CTATG AAACT GCTCT GG | 143 | EU_030283.2 |
| *INSR* | CTGCGTCACTTCACTGGCTA | TCATC TGCCT TGGCT TCAGG | 122 | XM_005654749.1 |
| *INS1* | TGGAT GATTC TGTGG TGGCC | CTGAT GGGGT TGGAG CAGTT | 124 | NM_001244489.1 |
| *PIK3* | GCTGT GCTGG ATATT GCGTG | GAGGA AGAGG CTTTG GGTCC | 141 | NM_001012956.2 |
| *GLU-2* | GACACGTTTTGGGTGTTCCG | GAGGCTAGCAGATGCCGTAG | 149 | NM_001097417.1 |
| *G6PC* | AAG CCA AGC GAA GGT GTG AGC | GGA ACG GGA ACC ACT TGC TGA G | 165 | NM_001113445 |
| *PCK1* | TCA GCA CGA CTC CAG CCT TCA | GCT CAA GCA GTC TGG GCA TTC T | 122 | NM_001123158 |
| *GSK3* | TTCAG TCCTG GCGAA CTCAC | TGAGG ATGAG GTGAG GGAGG | 102 | XM_003127173.3 |
| *GYS2* | TGGGAATTCTGTGGGAAGCC | TAGGTGCACTTGATGCAGGG | 110 | NM-001195511.1 |

*ANGPTL4*, angiopoietin-like 4; *PPAR-γ*, peroxisome proliferator-activated receptor gamma; *ACC*, acetyl-CoA carboxylase; *FAS*, fatty acid synthase; *CD36*, atty acid transporter CD36; *LPL*, lipoprotein lipase; *SREBP-1C*, sterol regulatory element binding protein 1C; *PRKAA1*, AMP activated alpha 1; *PRKAA2*, AMP activated alpha 2; *CPT-1B*, carnitine palmitoyltransferase 1 B; *PNPLA2*, adipose triglyceride lipase; *PGC-1α*, peroxisome proliferator-activated receptor gamma coactivator-1α; *FOX-1*, foxo1 forkhead box O1; *Sirt1*, silent information regulator 1; *INSR*, insulin receptor; *IRS1*, insulin receptor substrate 1; *PIK3*, phosphatidylinositol 3-kinase catalytic subunit type 3; *GLU-2*, glucose transporter 2; *G6PC*, glucose-6-phosphatase; *PCK1*, phosphoenolpyruvate carboxykinase 1; *GSK3*, glycogen synthase kinase 3; *GYS2*, glycogen synthase 2.
